# The long-term domiciliary oxygen therapy experience and needs in patients with chronic obstructive pulmonary disease: a qualitative meta-synthesis

**DOI:** 10.3389/fresc.2026.1694213

**Published:** 2026-02-25

**Authors:** Guannan Ma, Shiyan Yao, Huajuan Shen, Yongze Dong, Luchen Chen, Haixin Song, Yanyu Fang, Xiaoli Shen

**Affiliations:** 1 School of Public Health and Nursing, Hangzhou Normal University, Hangzhou, China; 2 Department of Nursing, Zhejiang Provincial People’s Hospital, Hangzhou, China; 3 School of Nursing, Zhejiang Chinese Medical University, Hangzhou, China; 4 Department of Nursing, Zhejiang Chinese Medical University Affiliated Third Hospital, Hangzhou, China

**Keywords:** chronic obstructive pulmonary disease, experience, long-term domiciliary oxygen therapy, meta-synthesis, qualitative research

## Abstract

To systematically evaluate and integrate the experience and perception of long-term domiciliary oxygen therapy in patients with Chronic Obstructive Pulmonary Disease (COPD). A systematic search of PubMed, Embase, Web of Science, CINAHL, ProQuest, Scopus, Cochrane Library, VIP Database, WanFang, CNKI, and CBM Database for qualitative studies on the experience of long-term domiciliary oxygen therapy in patients with COPD was conducted. The time limit was from the database's inception to 1 August 2025. The qualitative meta-synthesis was performed according to the Preferred Reporting Items for Systematic Reviews and Meta-Analyses (PRISMA) guidelines. A total of 9 papers were included, and 33 outcomes were distilled into nine categories and four integrated outcomes: Health benefits of Long-Term Oxygen Therapy for patients with COPD, the patients with COPD faced many challenges after Long-Term Oxygen Therapy, adaptation to Long-Term Oxygen Therapy in patients with COPD, and the demands of patients with COPD undergoing Long-Term Oxygen Therapy. Although long-term domiciliary oxygen therapy for patients with COPD brings certain health benefits, they also face a series of problems and challenges. In the future, healthcare professionals should pay attention to the experience of long-term domiciliary oxygen therapy for patients with COPD and actively address its adverse effects.

**Systematic Review Registration:**

https://www.crd.york.ac.uk/PROSPERO/view/CRD420251086510, PROSPERO CRD420251086510.

## 1 Introduction

Chronic Obstructive Pulmonary Disease (COPD), commonly referred to as chronic obstructive lung disease, is defined as a heterogeneous lung disorder (1, 2). According to the World Health Organization (WHO), COPD stands as the fourth leading cause of death globally and is projected to become the third leading cause by 2030. The prevalence of COPD in China is also considerable, with epidemiological survey data indicating a prevalence rate of approximately 8.2% among the population aged 40 and above. As the disease progresses, the pulmonary function of patients with COPD continuously deteriorates, with airflow limitation gradually worsening. Without timely intervention, it may eventually lead to chronic respiratory failure (3). The quality of life for patients with COPD is severely impacted while simultaneously imposing a substantial economic burden on both families and society.

Long-Term Oxygen Therapy (LTOT) is one of the crucial interventions for treating chronic persistent hypoxemia in patients with COPD and improving their prognosis. It has been demonstrated to correct hypoxemia, slow the deterioration of pulmonary function, increase the survival rate of patients with COPD, and enhance their quality of life (4–6). However, compliance with LTOT is suboptimal in clinical practice, with many patients failing to adhere to treatment for various reasons. Studies (7–9) have indicated that some patients with COPD experience physiological discomforts such as nasal dryness, dizziness, impaired taste, and smell, as well as negative emotional states, including depression and demoralization during LTOT. These issues diminish the experience and quality of LTOT for patients with COPD and negatively impact their rehabilitation outcomes.

Gaining an in-depth understanding of patients with COPD undergoing LTOT and identifying controllable factors can improve patient experiences and enhance their quality of life. In recent years, an increasing number of researchers have employed qualitative research methods to explore the experiences of patients with COPD with long-term oxygen therapy. From various perspectives, these studies have revealed patients' psychological, physiological, and social needs during LTOT. However, individual primary studies, influenced by different cultural backgrounds, methodological approaches, and values, cannot provide a comprehensive reflection of the LTOT experiences of patients with COPD. Moreover, few systematic reviews synthesize and integrate the findings of related qualitative studies. Meta-synthesis, as a method for comprehensive analysis of multiple qualitative studies, helps to distill more universal and representative conclusions. By conducting a Meta-synthesis of existing qualitative studies on patients' LTOT experiences with COPD, we can better understand patients' genuine feelings throughout the treatment process and provide comprehensive, systematic intervention measures for healthcare professionals. Therefore, this study aims to conduct a Meta-synthesis of existing qualitative research on patients' LTOT experiences with COPD, providing references for healthcare professionals to develop personalized LTOT support strategies.

## 2 Aim

(1) What are the perceptions and experiences (both positive and negative) of patients with Chronic Obstructive Pulmonary Disease regarding long-term oxygen therapy? (2) What recommendations related to long-term oxygen therapy can be derived from the included studies for further clinical practice, education, and research?

## 3 Methods

### 3.1 Study design

By analyzing the results of multiple independent qualitative studies, qualitative research meta-synthesis can distill a more thorough and profound understanding, uncovering the commonalities and differences in the research topic. This process provides systematic evidence and guidance for clinical practice, theoretical construction, and policy formulation, enhancing the universality and credibility of the research. This systematic review and meta-synthesis is reported according to the guidelines of Enhancing Transparency in Reporting the Synthesis of Qualitative Research (ENTREQ) (10) (Supplementary Appendix 1). The detailed study protocol is available on the PROSPERO website under the registration number CRD420251086510.

### 3.2 Search strategy

A computerized search was conducted in databases including PubMed, Embase, Web of Science, CINAHL, ProQuest, Scopus, Cochrane Library, VIP Journal Database, Wanfang Database, China National Knowledge Infrastructure (CNKI), and China Biomedical Literature Database (CBM), and the references of included studies were also traced. The time limit was from the database's inception to 1 August 2025. The search strategy was initially developed by two researchers (Guannan Ma and Shiyan Yao), both trained in systematic reviews, and subsequently peer-reviewed by an expert in evidence-based nursing (Huajuan Shen) to ensure its comprehensiveness and accuracy. The search terms were a combination of subject headings and free-text words. The search terms included: “Chronic Obstructive Lung Disease/Chronic Obstructive Pulmonary Diseases/COPD/Chronic Obstructive Airway Disease/Chronic Obstructive Pulmonary Disease/Chronic Airflow Obstructions/Pulmonary Disease, Chronic Obstructive”; “Oxygen Inhalation Therapy/Inhalation Therapy, Oxygen/Oxygen Inhalation Therapies/Therapy, Oxygen Inhalation/long term domiciliary oxygen therapy”; “Psychological/Experience/Needs/perception*/feeling*”; “qualitative research/qualitative study/qualitative methods/interview/phenomenon/grounded theory/ethnographic research”. The search strategy is presented in Supplementary Appendix 2.

### 3.3 Selection criteria

The primary studies were selected based on the study design, participants, phenomena of interest, and context. The inclusion criteria were as follows: (1) Study participants: Patients diagnosed with COPD according to clinical guidelines and receiving LTOT. (2) Phenomena of interest: The real experiences or perceptions of patients with COPD during LTOT. (3) Context: Long-term oxygen therapy was conducted in patients' homes. (4) Study design: Qualitative studies using phenomenology, grounded theory, ethnography, and ethnology, encompassing interviews, observation, and thematic analysis. Additionally, mixed-methods studies were considered, but only their qualitative components were included.

The exclusion criteria included: (1) Studies not published in Chinese or English. (2) Duplicate publications or studies with incomplete data. (3) Studies for which the full text could not be obtained. (4) Review articles, conference papers, and studies with low-quality evaluation.

### 3.4 Literature screening and data extraction

Two researchers (Guannan Ma and Shiyan Yao) independently conducted the literature screening and data extraction, following the inclusion and exclusion criteria. Firstly, a database was established in EndNote to identify duplicates, and the titles and abstracts of the literature were carefully reviewed to exclude studies that did not meet the inclusion criteria. The full texts of potentially eligible studies were then read to determine the final inclusion. In cases of discrepancy, consensus was reached through discussion or consultation with a third researcher.

Two researchers (Guannan Ma and Shiyan Yao) independently extracted the data using the JBI Qualitative Assessment and Review Instrument. The data extraction included the first author's name, publication year, country, research method, study participants, phenomena of interest, and main findings. If necessary, correspondence via email was initiated with the authors to obtain additional or missing information. The extracted text was input verbatim into Excel for management and analysis.

According to the JBI credibility evaluation criteria, the extracted raw data results were assessed as “clear,” indicating rational evidence without any ambiguity; “ambiguous,” meaning that results could be inferred from examples but lacked a clear connection between the results and the examples; and “unsupported,” indicating that the examples did not support the research findings. The convergent synthesis method from the JBI Center for Evidence Synthesis was used to collect the research results. Based on a thorough understanding of the 10 items in the qualitative research evaluation tool, the researchers repeatedly read, analyzed, and interpreted the connections and meanings of each research result, summarized similar results to form new categories, and then synthesized these categories to produce new integrated results.

### 3.5 Methodological quality assessment and evidence grading of literature

The methodological quality of the included literature was independently assessed by two researchers (Guannan Ma and Shiyan Yao) using the Joanna Briggs Institute (JBI) Critical Appraisal Checklist for Qualitative Research (11) developed by the JBI Evidence-Based Healthcare Center. The quality assessment checklist consists of 10 items, each rated as “yes,” “no,” “unclear,” or “not applicable.” When all 10 items are answered “yes,” the likelihood of bias is minimal and rated as A. If some of the quality criteria are met but not all, the possibility of bias is considered moderate and is designated as B. If all items are answered “no,” the possibility of bias is deemed high and classified as C. In the event of discrepancies in the assessment results, a third researcher was consulted to make a final decision. Ultimately, studies with quality assessment levels A and B were included.

### 3.6 Meta-synthesis

The meta-synthesis was conducted by two researchers (Guannan Ma and Shiyan Yao) trained in systematic, evidence-based nursing, using the thematic synthesis method developed by Thomas and Harden (12), which consists of three stages. The first stage involved line-by-line coding of the text, including abstracts, tables, figures, survey results, conclusions, supplementary files, and other relevant data. The second stage entailed comparing different codes, integrating the original multiple codes, creating new codes, and forming a hierarchical tree diagram. The third stage required a recursive analysis of the previously constructed “descriptive” themes, aiming to generate findings that transcend the original studies. This process might be iterative until the “analytical” themes produced are abstract enough to describe or explain all the “descriptive” themes. Analytical memos were used to document thoughts in detail as the first author familiarized with the data and coded, to achieve reflexivity. In cases of disagreement between the two researchers, a group meeting was held with the research team to communicate and analyze the underlying causes of the discrepancies. This process continued until a consensus was reached.

### 3.7 Evidence quality assessment

The ConQual system (13) was used to evaluate the quality grade of the synthesized evidence. Researchers comprehensively assessed the reliability and credibility of the evidence, grading it from high quality down in stages. (1) Reliability Assessment: This was based on the results of literature evaluation items 2, 3, 4, 6, and 7 from the JBI literature quality evaluation criteria. If 4–5 items were rated as “Yes,” no downgrade was applied; if 2–3 items were rated as “Yes,” the grade was downgraded by one level; if 0–1items were rated as “Yes,” the grade was downgraded by two levels. (2) Credibility Assessment: If all original research results included in the synthesized findings were rated as “Clear,” no downgrade was applied; if the results included both “Ambiguous” and “Clear,” the grade was downgraded by one level; if only “Ambiguous” results were included, the grade was downgraded by two levels; if the results included both “Ambiguous” and “Not Supported,” the grade was downgraded by three levels; if all results were “Not Supported,” the grade was downgraded by four levels.

### 3.8 Ethical considerations

This was a meta-synthesis of previously published literature and did not include human participants. It was therefore exempt from ethics committee review. However, not all the original studies included in our review explicitly stated the ethics review committee's approval or detailed the process of obtaining informed consent from participants. During our quality evaluation, we used the JBI Qualitative Research Assessment Tool. We found that two studies lacked any mention of ethics review, which may raise concerns about the protection of participants' rights. Nevertheless, we thoroughly evaluated all studies with the JBI tool. Despite their omission regarding ethics review, these two studies met our criteria in other crucial methodological aspects and were thus included. We argue that their inclusion provides a more comprehensive understanding of the topic, but readers should be cautious when interpreting their results due to the lack of ethical reporting. Future research should aim to be transparent in reporting all ethical considerations to strengthen the overall credibility and rigor of the studies.

## 4 Results

### 4.1 Search outcomes

**Figure 1:**
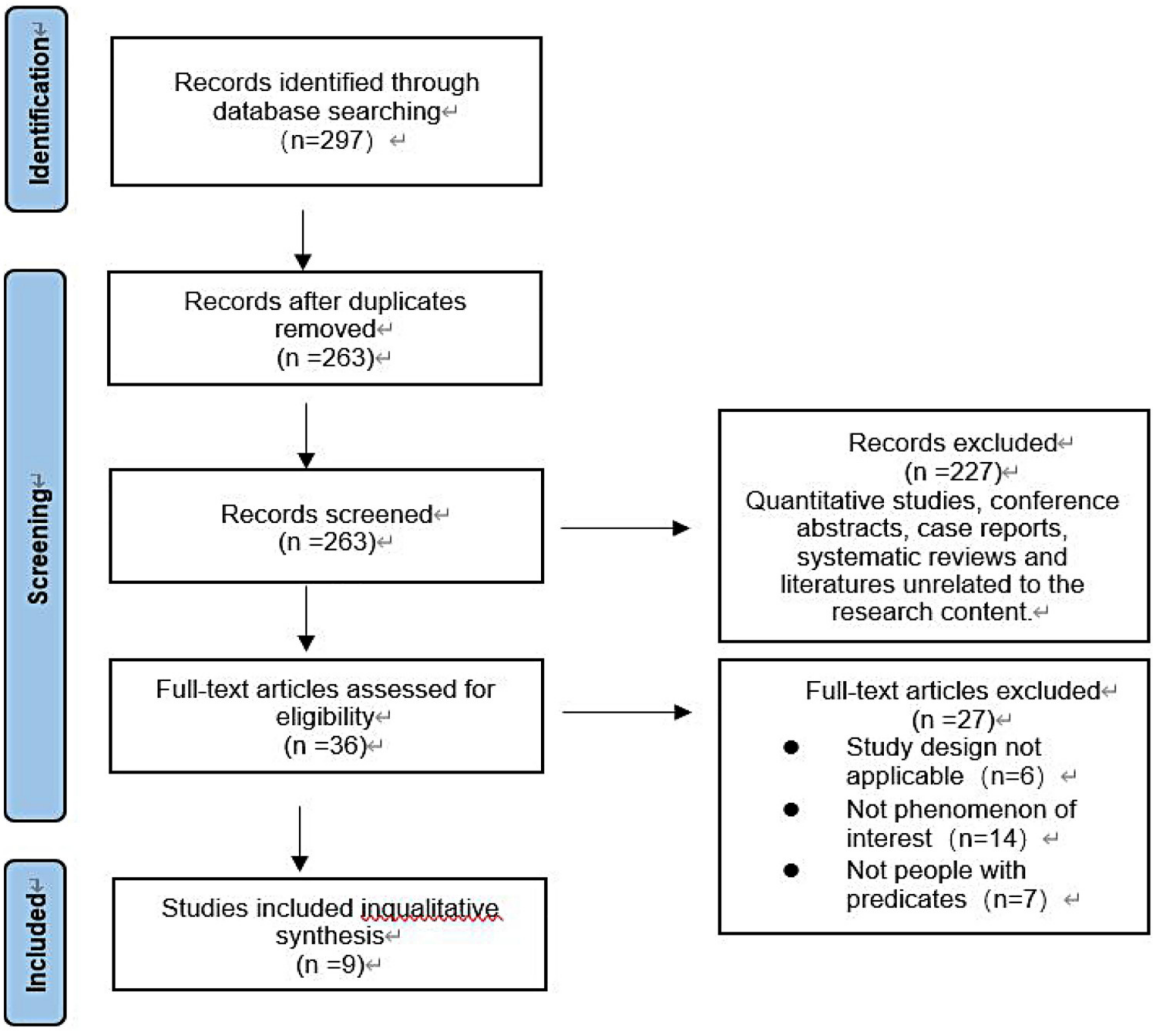
PRISMA flow diagram of the literature search and selection process.

The search strategy obtained 297 relevant articles. After deduplication, initial screening, secondary screening, and quality evaluation, 9 articles were included, 1 in Chinese and 8 in English. The literature screening process and results are shown in Figure 1.

### 4.2 Characteristics of the included studies

Nine studies were conducted in the United States, the United Kingdom, Denmark, Sweden, Brazil, and China from 2002 to 2022. The basic characteristics of the included literature are detailed in Supplementary Table S1. Additionally, all included studies met at least 7 of the quality evaluation criteria.

### 4.3 Quality appraisal of the included studies

The quality evaluation of the included studies indicated that all investigations adhered to most of the JBI qualitative research quality assessment items. However, few authors explicitly addressed their cultures and values. For a detailed quality assessment, refer to Supplementary Table S2.

### 4.4 Results of evidence quality assessment

The ConQual ratings for the four synthesized findings were “Moderate,” “Low,” “Moderate,” and “Moderate,” respectively (see Supplementary Table S3 for details).

### 4.5 Qualitative meta-synthesis

**Figure 2:**
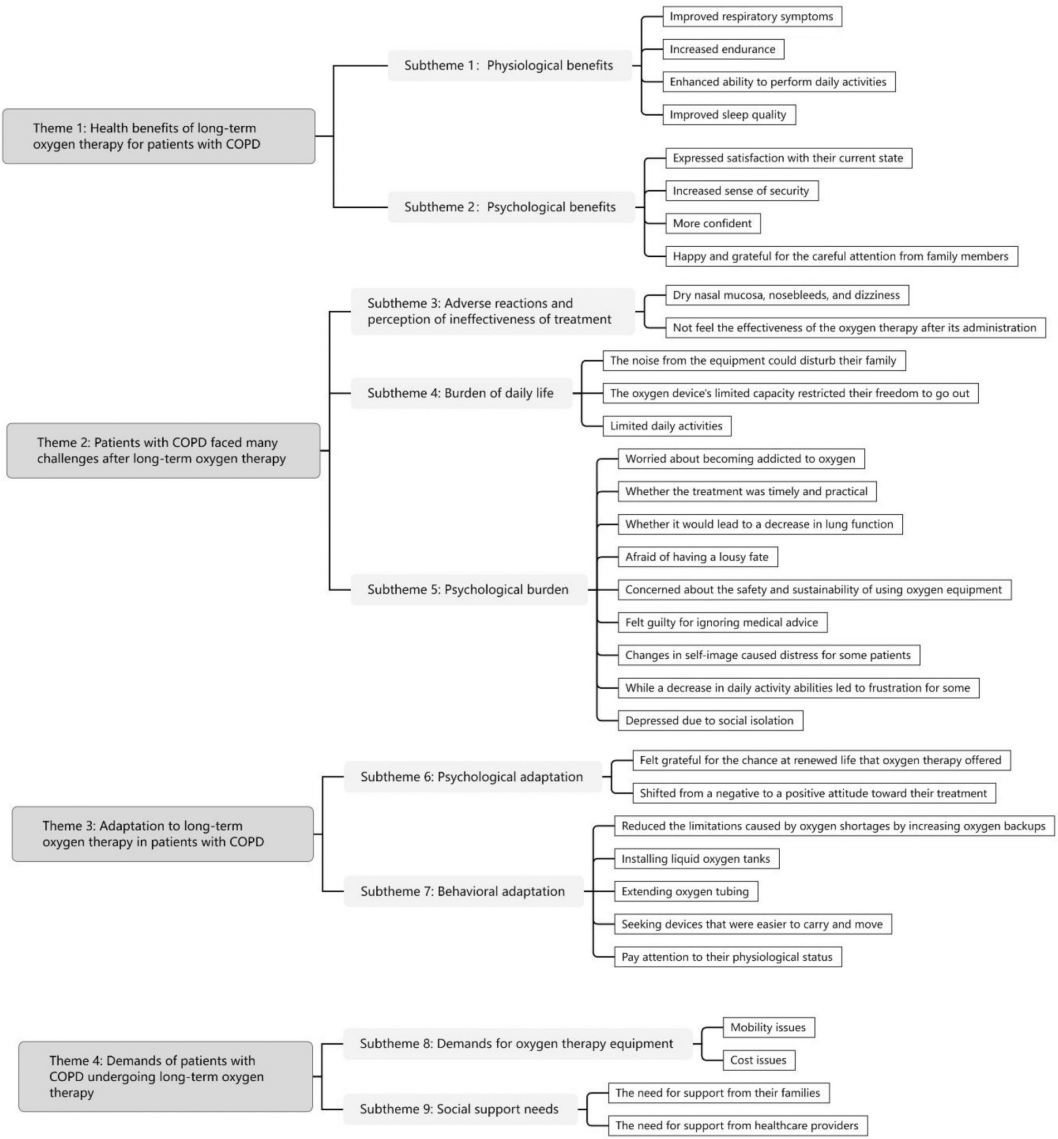
The integrated results.

In this study, we repeatedly read, compared, and interpreted the nine included studies. From this process, we extracted 33 findings, grouped them into nine categories, and ultimately synthesized these into four integrated findings. The integrated results are shown in Figure 2.

#### 4.5.1 Theme 1: health benefits of long-term oxygen therapy for patients with COPD

##### 4.5.1.1 Physiological benefits

Some patients with COPD reported improved respiratory symptoms following long-term oxygen therapy [“Compared to before, I felt like breathing was easier; I used to have to work hard to breathe”; “With the addition of a humidifier, coughing and expectoration became easier; I used to have a hard time getting it out” (14)], increased endurance [“After oxygen therapy, my endurance improved, and I could do things that were very strenuous before” (15)], enhanced ability to perform daily activities [“I could go to the park or eat out, do things I couldn't do before” (16)], and improved sleep quality [“After treatment, I slept better, more peacefully, and for longer than before” (14); “Before this, I could only sleep for 1–2 h at night, but now I could sleep through the night and feel relaxed” (16)]. The physiological benefits of long-term oxygen therapy for patients with COPD were significant.

##### 4.5.1.2 Psychological benefits

Some patients with COPD experienced relief from psychological issues as their physical symptoms subsided following long-term oxygen therapy, expressing satisfaction with their current state [“When I woke up in the morning, I felt good, my mood was better, I had more energy, I felt like a world champion, like I could do anything” (14)], an increased sense of security [“I felt safer with it; when I was short of breath, I didn't panic and feel afraid like I used to, this sense of security had an impact on me” (14)]; moreover, some patients became more confident due to a reduction in care dependency [“I felt like I'd regained some independence; I used to be completely dependent on others for help, and now I was very confident” (16)], and they were very happy and grateful for the careful attention from family members [“My husband said he'd take care of me until the last minute, and I was happy”; “I quit smoking 20 years ago, but when I smelled it, I still had a lot of desires; my wife used to smoke, but she quit for me, and I was very comforted” (17)].

#### 4.5.2 Theme 2: patients with COPD faced many challenges after long-term oxygen therapy

##### 4.5.2.1 Adverse reactions and perception of ineffectiveness of treatment

Although long-term oxygen therapy had been proven to correct hypoxemia, slow the deterioration of lung function, and increase the survival rate of patients with COPD, it also caused discomfort for some patients, such as dry nasal mucosa, nosebleeds, and dizziness [“Of course, there were some annoying effects, my nose was very dry and sometimes itchy” (18); “Sometimes I got nosebleeds, and it could even cause dizziness” (19)]. Additionally, some patients reported that they did not feel the effectiveness of the oxygen therapy after its administration [“I felt no difference when I used oxygen compared to when I didn't^”^ (15); “I hadn't gotten any benefit because I still couldn't breathe, that was the problem; I didn' think this was helpful for me, I didn't understand why I was still short of breath” (20)].

##### 4.5.2.2 Burden of daily life

Patients with COPD undergoing long-term oxygen therapy had to wear oxygen therapy equipment, which could impact their and their family members' quality of life. Some patients found that the noise from the equipment could disturb their family [“I found the noise annoying; I could get used to it at night, but my wife also had to put up with it” (14)]; at the same time, some patients believed that the oxygen device's limited capacity restricted their freedom to go out [“I was afraid it would run out quickly; I could see the dial go down, so I didn't dare leave the long-term oxygen therapy equipment, which in some ways restricted me and prevented me from being away for long periods” (20)]; and due to the heavy weight and large size of the equipment, it limited their daily activities [“I felt like I was locked up; I couldn't stand up and walk around, I had to stay next to the machine” (14); “My oxygen equipment weighed 5 pounds; it was heavy and bulky, making it difficult to maintain while cleaning the house, working in the yard, taking care of pets or children” (21)], thereby increasing the burden of daily life.

##### 4.5.2.3 Psychological burden

Some patients with COPD undergoing LTOT lacked security about the future, worrying about becoming addicted to oxygen [“I have gone from 1L to 4L, have I become addicted?” (18)], whether the treatment was timely and practical [“I worried that I wouldn't be able to use it in time when symptoms appeared, or that it would be ineffective even if I did use it” (22)], and whether it would lead to a decrease in lung function [“I couldn't rely completely on the oxygen equipment; I also had to let my lungs work. Carrying it around all the time would weaken my lung function, wouldn't it?” (15)]. Some patients were afraid of having a lousy fate [“When I needed to use oxygen, I thought of my brother lying in bed, which scared me” (22)]. Additionally, some patients expressed concerns about the safety and sustainability of using oxygen equipment [“I was terrified the oxygen cylinder would explode” (19); “I worried whether there would be enough oxygen for me to travel smoothly” (21)]. Some patients felt guilty for ignoring medical advice [“When I didn't follow the doctor's advice and my respiratory symptoms worsened, I felt guilty for not doing what I should have” (18)]. Moreover, changes in self-image caused distress for some patients [“I felt like everyone was staring at me, and I felt like a monster” (15)], while a decrease in daily activity abilities led to frustration for some [“I wanted to live by the sea, but now I couldn't move, how was I supposed to walk on the sand with oxygen? Ah” (15)]. Some patients became depressed due to social isolation [“My work stopped, I became socially isolated, I lost interest and desire, and sometimes I even wanted to commit suicide” (17)].

#### 4.5.3 Theme 3: adaptation to long-term oxygen therapy in patients with COPD

##### 4.5.3.1 Psychological adaptation

Despite the potential for long-term oxygen therapy to negatively impact patients' self-image, some patients still felt grateful for the chance at renewed life that oxygen therapy offered [“I didn't feel ashamed to use oxygen; it ensured my survival and hadn't brought anything else” (17)]. Some patients shifted from a negative to a positive attitude toward their treatment [“Before, I felt hideous, and many people on the street laughed at me when I walked by, but today the oxygen tube didn't bother me anymore because it was for my good, and I felt happier” (17); “She didn't complain about the discomfort caused by dryness anymore, and she didn't get up at night to drink water like she used to” (14)].

##### 4.5.3.2 Behavioral adaptation

Patients with COPD gradually adapted to the various changes brought about by long-term oxygen therapy. Some patients reduced the limitations caused by oxygen shortages by increasing oxygen backups [“When I went out for church activities, dining, or shopping, I placed oxygen equipment backups nearby in case of emergency” (15)], installing liquid oxygen tanks [“When I went out, I installed liquid oxygen in the car so that I could go camping and fishing” (15)], extending oxygen tubing [“I connected the oxygen tubing to the upstairs bedroom, so I didn't have to change the equipment frequently” (15)], and seeking devices that were easier to carry and move [“We negotiated with doctors, insurance companies, or oxygen suppliers until we got a lighter device” (15)]. Additionally, some patients began to monitor their physiological status [“I started to pay attention to my pulse, which sometimes reached 120 beats per minute, but after breathing oxygen, it started to decrease, indicating that oxygen therapy was effective” (16)].

#### 4.5.4 Theme 4: demands of patients with COPD undergoing long-term oxygen therapy

##### 4.5.4.1 Demands for oxygen therapy equipment

Some patients with COPD encountered pressing issues when using long-term oxygen therapy equipment, such as mobility issues [“It was too heavy; if there had been something lighter and more convenient, I would have used it, but the current equipment was completely unsuitable for my lifestyle” (20)], and cost issues [“My oxygen supplier said that insurance wouldn't cover them; they were too expensive, and I couldn't afford them” (21)].

##### 4.5.4.2 Social support needs

Social support was crucial for patients’ recovery and adherence to oxygen therapy. Some patients expressed the need for support from their families [“When I needed to receive oxygen therapy, my living space was greatly reduced, and I needed family help to bring the oxygen cylinder in” (19)], as well as from healthcare providers [“I bought the oxygen equipment from my doctor, but when I went out of the house, I didn't know when to use it, and I couldn't remember any instructions” (18)].

## 5 Discussion

### 5.1 The included literature was of high quality

All nine studies included in this research met at least 7 quality evaluation criteria and were thus included. Therefore, in the ConQual rating, the reliability of all four synthesized findings was downgraded to “Moderate.” In the credibility evaluation of the original research findings, two results were rated “Ambiguous” due to a lack of clear connection between the results and their exemplars. Consequently, in the ConQual rating, the credibility of “Synthesized Finding 2” was downgraded to “Moderate.” Ultimately, the ConQual ratings for the four synthesized findings were “Moderate,” “Low,” “Moderate,” and “Moderate,” respectively, which hold some value for the strength of recommendations for clinical practice.

### 5.2 Attention to the physical and psychological experiences of patients with COPD undergoing long-term oxygen therapy and improving their adverse experiences

The results of this study indicated that while long-term oxygen therapy for patients with COPD brought certain health benefits, it also brought about various physiological and psychological discomforts, such as dry and bleeding nasal mucosa, dizziness, worry, fear, depression, and distress. This was in alignment with the findings of Van et al. (23) and may have contributed to the low compliance with long-term oxygen therapy. This suggests that healthcare professionals should while focusing on patients' health benefits, provide personalized guidance based on their specific problems to reduce adverse experiences and enhance patient comfort. For patients with insufficient knowledge and skills in oxygen therapy, healthcare professionals guide them and their caregivers in selecting appropriate oxygen delivery methods, flow rates, and therapy durations to ensure adequate and safe oxygenation. Research (24) indicated that High-Flow Nasal Cannula Oxygen Therapy (HFNC) delivered a heated, humidified gas flow, keeping the airway epithelium adequately moisturized, improving mucus clearance, and reducing irritation. Furthermore, in comparison with Conventional Oxygen Therapy, HFNC was perceived as more comfortable, reduced the risk of irritation and ulcers, and was better tolerated by patients. For patients who perceived the therapy as ineffective, healthcare professionals would advise them to seek prompt medical attention to avoid any negative impact on prognosis from changes in their condition. For patients who lacked a sense of security and experienced worry and fear, healthcare professionals would provide enhanced safety education for oxygen therapy, such as prohibiting smoking and open flames and using electronic devices to ensure safety during long-term oxygen therapy (25). Research (26) has demonstrated that Cognitive Behavioral Therapy (CBT) can significantly alleviate anxiety and depression in patients with COPD. For patients experiencing depression and frustration due to an impaired self-image, limited freedom, and social isolation, healthcare professionals should employ CBT to guide them in identifying and challenging maladaptive cognitions. Concurrently, implementing progressive behavioral activation programs to encourage gradual social re-engagement can break the vicious cycle of depression and isolation through positive action.

### 5.3 Assessing patients' motivation for self-adaptation to long-term oxygen therapy and assisting in their psychological and behavioral self-adjustment

Self-adaptation was defined as the individual's capacity to modify their perspectives, thoughts, and behaviors to achieve a goal, and it was considered a vital skill for adapting to one's environment. This ability could enhance subjective initiative and self-management skills (27). The integrated results of this study indicated that, despite the potential for long-term oxygen therapy to cause various discomforts, some patients could maintain a positive outlook and enhance their self-adaptation by adding backups, installing liquid oxygen tanks, and extending tubing. Therefore, it was possible to draw upon the extroverted group therapy method (28) by forming groups consisting of patients with both positive and negative coping strategies. Patients with positive coping strategies could share their experiences with long-term oxygen therapy, thereby helping those with negative coping strategies learn optimism, acceptance, and proactive coping. Furthermore, studies (29, 30) have demonstrated that nursing interventions grounded in empowerment theory can enhance patients with COPD's quality of life, improve their self-efficacy, and reduce negative emotions. Healthcare professionals should closely monitor patients' psychological and behavioral changes, assess motivation for self-adaptation to long-term oxygen therapy in patients with COPD, and use the extroverted group therapy method and nursing interventions grounded in empowerment theory to promote self-adaptation and improve patients' health outcomes.

### 5.4 Emphasizing the long-term oxygen therapy needs of patients with COPD and improving the social support system

The integrated results of this study revealed that patients with COPD undergoing long-term oxygen therapy faced pressing issues, such as the performance and cost of oxygen therapy equipment and insufficient external support. These findings were similar to those of Wang Yan (31), and these issues could affect patients' experience with long-term oxygen therapy and their health outcomes. Therefore, it was essential to address these existing problems promptly and to build a diversified social support system to meet patients' urgent needs. For instance, given the heavy, noisy, and expensive nature of oxygen therapy equipment, it was suggested that product developers should actively improve the devices, developing more practical, convenient, and quiet models to enhance patients' experience. Long-term oxygen therapy was a crucial treatment for patients with COPD, but the economic burden it created was also a significant factor that may have hindered long-term use. Therefore, it was worth considering whether home-use ventilators and oxygen generators could be included in medical insurance. Moreover, relevant guidelines suggested that healthcare professionals should provide suitable and effective equipment based on the patient's characteristics, preferences, and needs (32). Mobile oxygen devices, such as wearable oxygen systems or portable oxygen generators, were recommended for frequently active patients. Healthcare professionals should have provided comprehensive education to all patients with COPD undergoing long-term oxygen therapy and their caregivers, ensuring they understood the necessity and importance of home oxygen therapy. They should also have guided them in mastering the operation process and precautions, ensuring correct use, and conducting regular follow-ups to address issues that may have arisen during use. Additionally, healthcare professionals should have informed patients with COPD’ caregivers about the importance of emotional support within the family, encouraging them to listen to the patient's inner demands, understand and empathize with them, and help them cope with psychological distress (33), thereby enhancing compliance with long-term oxygen therapy. The construction of a robust primary healthcare team was also crucial to forming a comprehensive management system for home oxygen therapy and building a support system for patients with COPD undergoing long-term oxygen therapy.

## 6 Limitations

This study has several limitations. First, although the included studies originated from multiple countries and regions, their limited number and a content analysis revealed that none explicitly examined the issue from the perspective of values and cultural backgrounds. Consequently, we were unable to determine cross-cultural differences in long-term home oxygen therapy for COPD patients, nor could we investigate the impact of these cultural variations on their experience. This may limit the generalizability of our findings. Second, all included literature was assessed as B-grade quality, which may have introduced bias into the synthesis results. Third, our search was restricted to studies published in indexed journals in both Chinese and English, and we excluded grey literature and dissertations, which may have led to publication bias. Fourth, the included studies span a wide time frame, with a scarcity of literature from the last three years. Consequently, while newer oxygen therapy devices and improved treatment measures have emerged, there is a lack of investigation into patients' current experiences.

Future research should not only delve deeper into the role of cultural and geographical factors but also actively explore the tangible impact of the latest home oxygen therapy measures and guidelines on patient experience and treatment adherence.

## 7 Conclusion

This study conducted a meta-synthesis to systematically interpret the experiences of patients with COPD undergoing long-term oxygen therapy. The results showed that while long-term oxygen therapy for Patients with COPD brings certain health benefits, it also presents numerous challenges and issues. In the future, healthcare professionals should strengthen health education for patients and caregivers on long-term oxygen therapy, build a diversified social support system, focus on addressing the adverse experiences of long-term oxygen therapy, enhance psychological support for patients, alleviate their physical and mental burdens, and improve the comfort level of patients undergoing long-term oxygen therapy.

## Data Availability

The original contributions presented in the study are included in the article/Supplementary Material, further inquiries can be directed to the corresponding author.
