# Supplementary material for: The long-term domiciliary oxygen therapy experience and needs in patients with chronic obstructive pulmonary disease: a qualitative meta-synthesis
Source: Front Rehabil Sci. 2026 Feb 25;7:1694213. doi: 10.3389/fresc.2026.1694213 (PMC12976016; doi:10.3389/fresc.2026.1694213)
Supplement: Supplementary file 2 [file Table2.docx]

**China National Knowledge Infrastructure (CNKI)**

#1慢阻肺 + 慢性阻塞性肺疾病 + copd

#2氧疗 + 长期氧疗 + 家庭氧疗 + 长期居家氧疗

#3体验 + 经历 + 感受 + 需求 + 需要 + 态度

#4质性研究 + 现象学 + 扎根理论 + 民族志 + 人种学

#5 #1 + #2 + #3 + #4

**Wanfang Database**

主题:(慢阻肺 OR 慢性阻塞性肺疾病 OR copd) AND 主题:(氧疗 OR 长期氧疗 OR 家庭氧疗 OR 长期居家氧疗) AND 主题:(体验 OR 经历 OR 感受 OR 需求 OR 需要 OR 态度) AND 主题:(质性研究 OR 现象学 OR 扎根理论 OR 民族志 OR 人种学)

**VIP Journal Database**

题名或关键词:(慢阻肺 OR 慢性阻塞性肺疾病 OR copd) AND 题名或关键词:(氧疗 OR 长期氧疗 OR 家庭氧疗 OR 长期居家氧疗) AND 题名或关键词:(体验 OR 经历 OR 感受 OR 需求 OR 需要 OR 态度) AND 题名或关键词:(质性研究 OR 现象学 OR 扎根理论 OR 民族志 OR 人种学)

**China Biomedical Literature Database (CBM)**

常用字段:(慢阻肺 OR 慢性阻塞性肺疾病 OR copd) AND 常用字段:(氧疗 OR 长期氧疗 OR 家庭氧疗 OR 长期居家氧疗) AND 常用字段:(体验 OR 经历 OR 感受 OR 需求 OR 需要 OR 态度) AND 常用字段:(质性研究 OR 现象学 OR 扎根理论 OR 民族志 OR 人种学)

**PubMed**

#1 ("Chronic Obstructive Lung Disease"[Title/Abstract]) OR ("Chronic Obstructive Pulmonary Diseases"[Title/Abstract]) OR (COPD[Title/Abstract]) OR ("Chronic Obstructive Airway Disease"[Title/Abstract]) OR ("Chronic Obstructive Pulmonary Disease"[Title/Abstract]) OR ("Chronic Obstructive Airway Disease"[Title/Abstract]) OR ("Chronic Airflow Obstructions"[Title/Abstract]) OR ("Pulmonary Disease, Chronic Obstructive"[Mesh])

#2 ("Oxygen Inhalation Therapy"[Mesh]) OR ("Inhalation Therapy, Oxygen"[Title/Abstract]) OR ("Oxygen Inhalation Therapies"[Title/Abstract]) OR ("Therapy, Oxygen Inhalation"[Title/Abstract]) OR（"long term domiciliary oxygen therapy"[Title/Abstract])

#3 (Psychological[Title/Abstract]) OR (Experience[Title/Abstract]) OR (Needs[Title/Abstract]) OR (perception*[Title/Abstract]) OR (feeling*[Title/Abstract])

#4("qualitative research"[Title/Abstract]) OR ("qualitative study"[Title/Abstract]) OR ("qualitative methods"[Title/Abstract]) OR (interview[Title/Abstract]) OR (phenomenon[Title/Abstract]) OR ("grounded theory"[Title/Abstract]) OR ("ethnographic research"[Title/Abstract])

#5 #1 AND #2 AND #3 AND #4

**Wos**

#1 TS=("Chronic Obstructive Lung Disease" OR "Chronic Obstructive Pulmonary Diseases" OR “COPD” OR "Chronic Obstructive Airway Disease" OR "Chronic Obstructive Pulmonary Disease" OR "Chronic Obstructive Airway Disease" OR "Chronic Airflow Obstructions" OR "Pulmonary Disease, Chronic Obstructive")

#2 TS=("Oxygen Inhalation Therapy" OR "Inhalation Therapy, Oxygen" OR "Oxygen Inhalation Therapies" OR "Therapy, Oxygen Inhalation" OR "long term domiciliary oxygen therapy")

#3 TS=(“Psychological” OR “Experience” OR “Needs” OR “perception*” OR “feeling*”)

#4 TS=("qualitative research" OR "qualitative study" OR "qualitative methods" OR “interview” OR “phenomenon” OR "grounded theory" OR "ethnographic research")

#5 #1 AND #2 AND #3 AND #4

**Embase**

#1 'Chronic Obstructive Lung Disease':ab,ti OR 'Chronic Obstructive Pulmonary Diseases':ab,ti OR 'COPD':ab,ti OR 'Chronic Obstructive Airway Disease':ab,ti OR 'Chronic Obstructive Pulmonary Disease':ab,ti OR 'Chronic Obstructive Airway Disease':ab,ti OR 'Chronic Airflow Obstructions':ab,ti OR 'Pulmonary Disease, Chronic Obstructive'/exp

#2 'Oxygen Inhalation Therapy'/exp OR 'Inhalation Therapy, Oxygen':ab,ti OR 'Oxygen Inhalation Therapies':ab,ti OR 'Therapy, Oxygen Inhalation':ab,ti OR 'long term domiciliary oxygen therapy':ab,ti

#3 'Psychological':ab,ti OR 'Experience':ab,ti OR 'Needs':ab,ti OR 'perception*':ab,ti OR 'feeling*':ab,ti

#4 'qualitative research':ab,ti OR 'qualitative study':ab,ti OR 'qualitative methods':ab,ti OR 'interview':ab,ti OR 'phenomenon':ab,ti OR 'grounded theory':ab,ti OR 'ethnographic research':ab,ti

#5 #1 AND #2 AND #3 AND #4

**PROQUEST**

#1 ("Chronic Obstructive Lung Disease" OR "Chronic Obstructive Pulmonary Diseases" OR “COPD” OR "Chronic Obstructive Airway Disease" OR "Chronic Obstructive Pulmonary Disease" OR "Chronic Obstructive Airway Disease" OR "Chronic Airflow Obstructions" OR "Pulmonary Disease, Chronic Obstructive")

#2 ("Oxygen Inhalation Therapy" OR "Inhalation Therapy, Oxygen" OR "Oxygen Inhalation Therapies" OR "Therapy, Oxygen Inhalation" OR "long term domiciliary oxygen therapy")

#3 (“Psychological” OR “Experience” OR “Needs” OR “perception*” OR “feeling*”)

#4 ("qualitative research" OR "qualitative study" OR "qualitative methods" OR “interview” OR “phenomenon” OR "grounded theory" OR "ethnographic research")

#5 #1 AND #2 AND #3 AND #4

**Scopus**

#1 TITLE-ABS-KEY("Chronic Obstructive Lung Disease" OR "Chronic Obstructive Pulmonary Diseases" OR “COPD” OR "Chronic Obstructive Airway Disease" OR "Chronic Obstructive Pulmonary Disease" OR "Chronic Obstructive Airway Disease" OR "Chronic Airflow Obstructions" OR "Pulmonary Disease, Chronic Obstructive")

#2 TITLE-ABS-KEY("Oxygen Inhalation Therapy" OR "Inhalation Therapy, Oxygen" OR "Oxygen Inhalation Therapies" OR "Therapy, Oxygen Inhalation" OR "long term domiciliary oxygen therapy")

#3 TITLE-ABS-KEY( “Psychological” OR “Experience” OR “Needs” OR “perception*” OR “feeling*”)

#4 TITLE-ABS-KEY("qualitative research" OR "qualitative study" OR "qualitative methods" OR “interview” OR “phenomenon” OR "grounded theory" OR "ethnographic research")

#5 #1 AND #2 AND #3 AND #4

**Cinahl**

#1 TI ("Chronic Obstructive Lung Disease" OR "Chronic Obstructive Pulmonary Diseases" OR “COPD” OR "Chronic Obstructive Airway Disease" OR "Chronic Obstructive Pulmonary Disease" OR "Chronic Obstructive Airway Disease" OR "Chronic Airflow Obstructions" OR "Pulmonary Disease, Chronic Obstructive") OR AB ("Chronic Obstructive Lung Disease" OR "Chronic Obstructive Pulmonary Diseases" OR “COPD” OR "Chronic Obstructive Airway Disease" OR "Chronic Obstructive Pulmonary Disease" OR "Chronic Obstructive Airway Disease" OR "Chronic Airflow Obstructions" OR "Pulmonary Disease, Chronic Obstructive") OR MH ("Pulmonary Disease, Chronic Obstructive")

#2 TI ("Oxygen Inhalation Therapy" OR "Inhalation Therapy, Oxygen" OR "Oxygen Inhalation Therapies" OR "Therapy, Oxygen Inhalation" OR "long term domiciliary oxygen therapy") OR AB ("Oxygen Inhalation Therapy" OR "Inhalation Therapy, Oxygen" OR "Oxygen Inhalation Therapies" OR "Therapy, Oxygen Inhalation" OR "long term domiciliary oxygen therapy") OR MH ("Oxygen Inhalation Therapy")

#3 TI (“Psychological” OR “Experience” OR “Needs” OR “perception*” OR “feeling*”) OR AB (“Psychological” OR “Experience” OR “Needs” OR “perception*” OR “feeling*”)

#4 TI ("qualitative research" OR "qualitative study" OR "qualitative methods" OR “interview” OR “phenomenon” OR "grounded theory" OR "ethnographic research") OR AB ("qualitative research" OR "qualitative study" OR "qualitative methods" OR “interview” OR “phenomenon” OR "grounded theory" OR "ethnographic research")

#5 #1 AND #2 AND #3 AND #4

**Cochrane Library**

#1 ("Chronic Obstructive Lung Disease" OR "Chronic Obstructive Pulmonary Diseases" OR “COPD” OR "Chronic Obstructive Airway Disease" OR "Chronic Obstructive Pulmonary Disease" OR "Chronic Obstructive Airway Disease" OR "Chronic Airflow Obstructions" OR "Pulmonary Disease, Chronic Obstructive"):ti,ab,kw OR ("Pulmonary Disease, Chronic Obstructive")

#2 ("Oxygen Inhalation Therapy") OR ("Oxygen Inhalation Therapy" OR "Inhalation Therapy, Oxygen" OR "Oxygen Inhalation Therapies" OR "Therapy, Oxygen Inhalation" OR "long term domiciliary oxygen therapy"):ti,ab,kw

#3 (“Psychological” OR “Experience” OR “Needs” OR “perception*” OR “feeling*”):ti,ab,kw

#4 ("qualitative research" OR "qualitative study" OR "qualitative methods" OR “interview” OR “phenomenon” OR "grounded theory" OR "ethnographic research"):ti,ab,kw

#5 #1 AND #2 AND #3 AND #4
